# Efficient deep neural networks for cancer detection on histopathology combining attention and image downsampling

**DOI:** 10.1038/s41598-025-20954-2

**Published:** 2025-10-22

**Authors:** Miguel Socolovsky, Alberto López, Joel K. Greenson, Gad Rennert, Stephen B. Gruber, Victor Moreno

**Affiliations:** 1 Oncology Data Analytics Program, Catalan Institute of Oncology (ICO), Barcelona, Spain; 2 Department of Clinical Sciences, Faculty of Medicine and Health Sciences and Universitat de Barcelona Institute of Complex Systems (UBICS), University of Barcelona (UB), Barcelona, Spain; 3 Colorectal Cancer Group, ONCOBELL Program, Institut de Recerca Biomedica de Bellvitge (IDIBELL), Barcelona, Spain; 4 Department of Pathology, University of Michigan, Ann Arbor, MI USA; 5 Carmel Medical Center, Haifa, Israel; 6 Center for Precision Medicine, City of Hope, Duarte, California USA; 7 Consortium for Biomedical Research in Epidemiology and Public Health (CIBERESP), Madrid, Spain

**Keywords:** Cancer imaging, Colorectal cancer

## Abstract

Pathology diagnosis of colorectal cancer is time-consuming and requires a high level of expertise. However, it is an essential step towards establishing the adequate treatment. The need to analyse a large number of these histopathological images calls for automatic tools capable of aiding pathologists in this arduous task. Deep learning techniques, together with the wealth of data available nowadays, provide a promising candidate for such job. Adopting state-of-the-art artificial intelligence algorithms, we developed a model to accurately detect colorectal cancer in digitalised histopathological whole-slide images. Our end-to-end approach uses the principles of multiple-instance learning combined with deep convolutional neural networks in order to fully leverage the information contained within each image and make robust predictions at the patient’s level. The model also allows to highlight the areas in the slide most likely to harbour tumour tissue. Given the finite computational resources available, working at maximum resolution can be detrimental. Therefore, we explored the impact of lowering the working image resolution. The algorithms were trained and validated on a subset of more than 1300 patients of the Molecular Epidemiology of Colorectal Cancer study with histopathology images available. These images gave rise to $>10^5$ tiles of $256\times 256$ pixels each. Once we identified the best-performing model we put it to the test on images from The Cancer Genome Atlas. We obtained the best outcomes working at 4 μm/pix, achieving the following metrics on the test dataset: F1-Score of 0.96, a Matthews correlation coefficient of 0.92 and an area under the receiver operating characteristic curve of 0.99. These results are exceptional and prove that computational costs can be reduced while keeping the performance up to standard.

## Introduction

Colorectal cancer (CRC) is among the most frequent types of cancer and was the second most common cause of cancer mortality in 2020^1^. Early and accurate detection is key to improve the treatment effectiveness and the survival rate. Currently, CRC diagnosis demands visual examination by specialised pathologists, which increases the workload in areas where the incidence of this disease is high^2^. Besides, the complexity of the task often leads to a certain degree of subjectivity, giving rise to potential inter-observer disagreement. This calls for better tools to aid pathologists to effectively cope with the increasing demand of this task. Similarly, we need ways to make the diagnosis through histopathological imaging more deterministic and less subject to the human factor.

The digitalisation of hematoxylin & eosin (H&E)-stained whole-slide images (WSIs) has allowed for the accumulation of vast amounts of data over the past years^3^. Artificial intelligence (AI) techniques allow us to exploit this wealth of data in an unprecedented way. The field of machine learning applied to histopathology has grown in an accelerated manner in recent years^4–6^, with applications to lung^7,8^, skin^9^, breast^10,11^ and prostate^12,13^ cancer, among others. Although still emerging and constantly changing over time, it is an extremely promising field of study with a large potential in medicine.

One of the main challenges in this field is to gather a sufficiently large and general collection of CRC images. Even though the volume of data is larger than ever before, it is scattered between many sources, e.g. hospitals or laboratories. Along these lines, there is a large variability among datasets: magnification disparity^14^, staining fluctuations^15^ and the use of different scanners are some factors that contribute to the image batch effects^16^. Furthermore, we lack a unified scheme for labelling, storing and sharing the data. Therefore, the potential of these algorithms remains dormant as the main limiting factor is the access to high-quality data. Additionally, WSIs tend to be large in size, imposing yet another stumbling block to unveiling the full potential of AI in anatomical pathology. High-performance resources, such as large graphics processing units (GPUs), are not always available and efforts are being made to reduce computational costs and make AI solutions available to everyone.

In this study we focused on the aforementioned challenges: generalisation power and reduction of computational demand. Furthermore, we aimed to develop a unique pipeline, which improves its optimisation and, therefore, its performance. Firstly, we tried to obtain a viable WSI classifier for CRC, which allowed us to detect the presence of tumour tissue and the location of it. We put special effort in making this model as general as possible, for this matter we used different datasets for training and testing of the models. Secondly, we analysed the impact of image resolution on the classification accuracy. This is important because reducing the size of the images alleviates significantly the computational constraints, and at the same time, allows for the use of images obtained with lower resolution. Ultimately, we aimed to sample more efficiently the WSIs and speed up both training and inference. We therefore analysed four different resolution levels: $R=0$ (2 μm/pix), $R=1$ (4 μm/pix), $R=2$ (8 μm/pix) and $R=3$ (16 μm/pix).

## Data and methods

### Cohort

**Fig. 1:**
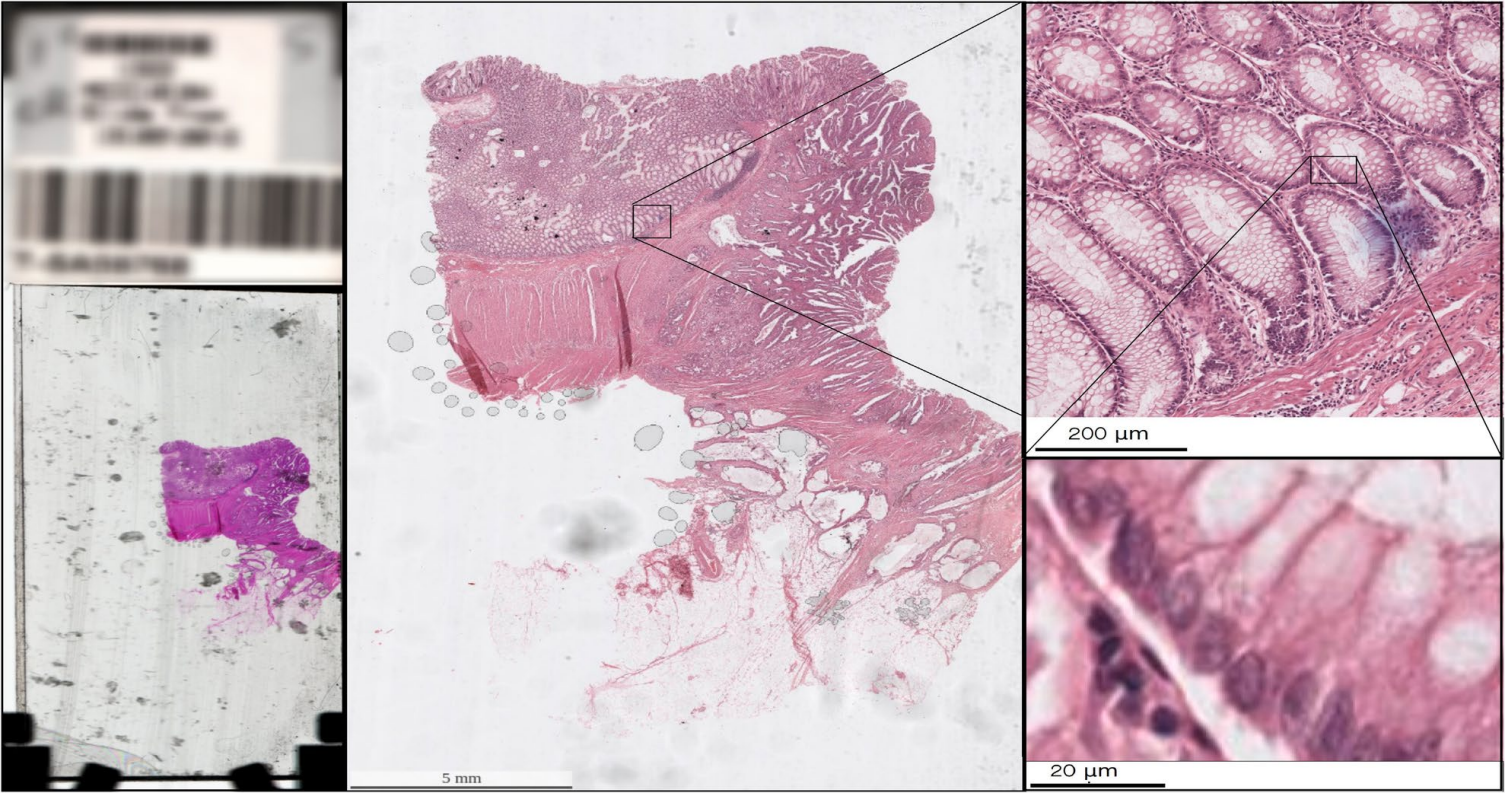
Colorectal histological slide from the MECC dataset. The first image on the left corresponds to the original slide of dimensions $25 \times 75~\text {mm}$. The largest and central one corresponds to the actual WSI, as we use it in this work. The top-right image is a zoom-in of approximately $600 \times 800$ µm. The image on the bottom-right is a close-up of about $60 \times 60$ µm where we can start to see the individual pixels. It is worth noting that the apparent colour discrepancy between the full WSI image and the rest arises from the former being a thumbnail, generated by a different machine. Staining remains consistent across all magnification levels within the dataset.

The Molecular Epidemiology of Colorectal Cancer (MECC) is a population-based case-control study of incident CRC cases in northern Israel during the time period between 1998 and 2016. For this study we used the histopathology data collected at diagnosis, reviewed and annotated by an expert pathologist (JKG). These were $\sim \!\!1317$ digitalised H&E WSIs. WSIs in this set had a magnification of $40\times$ and a raw resolution of $0.25$ μm/pix (see Fig. 1 for an example). Such images typically contain of the order of $10^9$ RGB pixels, which is equivalent to $\sim \!\!1.5~\text {GB}$ per image. Finally, $53\%$ of the samples were classified as dominated by tumour tissue, $29\%$ as mixture of both normal and tumour and the remaining $18\%$ contained only normal tissue. Hence, $82\%$ of the samples contained tumour while $18\%$ did not. The MECC dataset, used for training our model, is not publicly available but is available from SBG on reasonable request.

The Cancer Genome Atlas (TCGA) is a study that attempts to molecularly characterise a large sample of primary cancer, spanning 33 different cancer types. From this collection we obtained 1349 H&E WSIs of CRC, both magnification and resolution are consistent with those from the MECC. From these images, $91\%$ were labelled as tumour and the remaining $9\%$ as normal. Images were downloaded from the public repository (https://portal.gdc.cancer.gov/).

The EPICO study was approved by the Bellvitge Hospital Ethics Committee (Protocol Number PR132/21). The studies were conducted in accordance with the local legislation and institutional requirements. The participants provided their written informed consent to participate in this study.

### Defects and biases in the training data

**Fig. 2:**
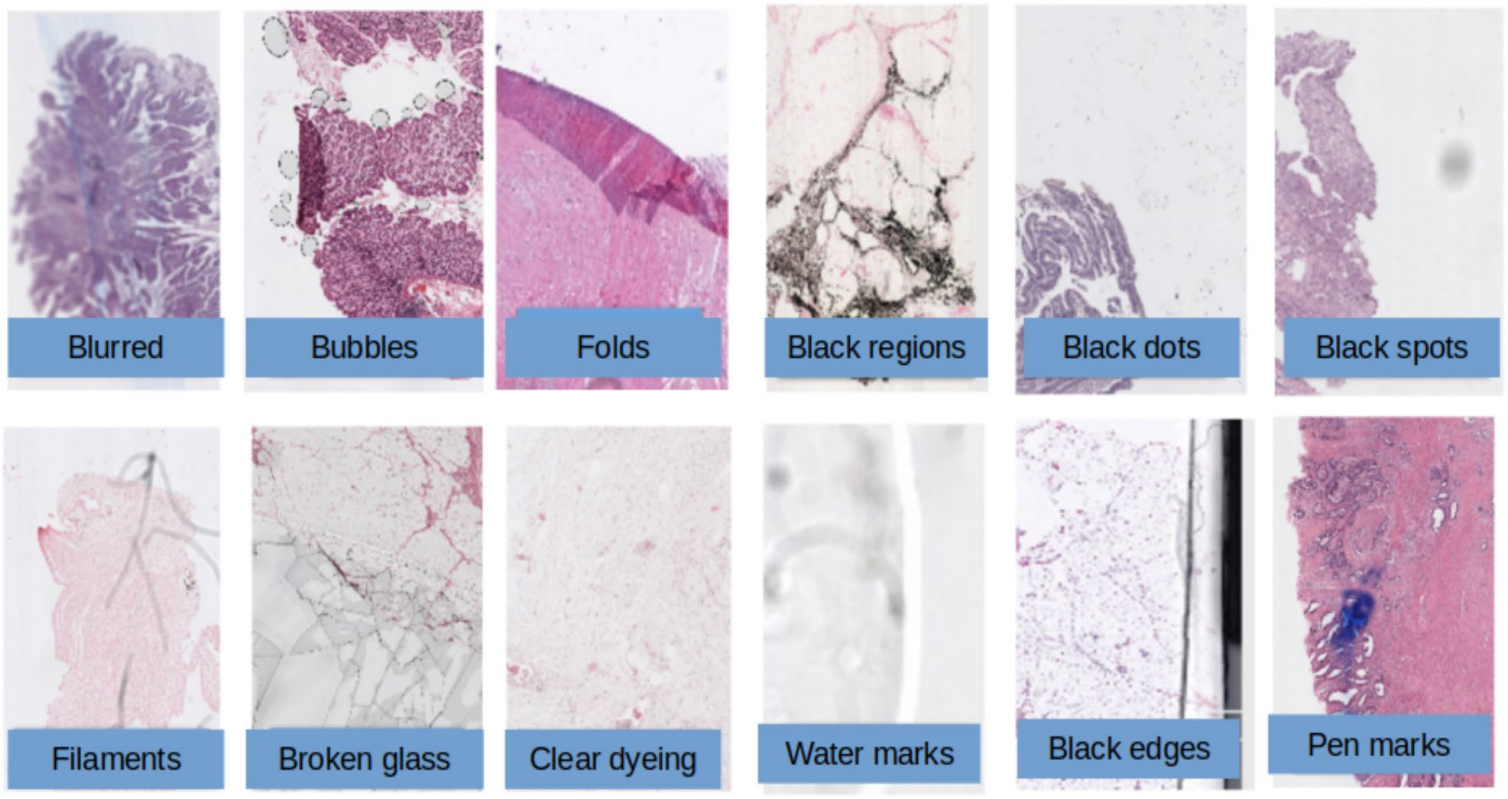
Examples of the most common defects found on the WSIs. These may include damaged samples, scanning defects, artefacts or traces of manual annotations. These need to be assessed to ensure they do not represent a significant bias in the training dataset.

WSIs often present a number of defects and artefacts. We identified the following ones: blurred areas, air bubbles, black regions, black dots, black spots, filaments, broken glass, clear dyeing, watermarks, black edges, pen marks and folds (see Fig. 2). Although defective WSIs should be removed if detected, we expect a small fraction of them to be leaked into the final dataset. Furthermore, some of the artefacts appear naturally in a significant fraction of the samples. Therefore, we need to ensure such features do not originate significant biases in the training dataset. For example, if tumours slides had previously been annotated, leaving occasional pen mark traces only on WSIs containing tumour tissue. This would correspond to a harmful type of bias given that a model could wrongly rely on the presence of pen marks to predict the presence of cancer.

Thus, we analysed the recurrence of the artefacts mentioned above and their distribution across classes (tumour vs normal), see Table 1. Furthermore, we performed a Z-test to determine if the difference in the proportions of these defects across class is significant. We only apply the statistical test when the number of slides affected was at least 15. Additionally, we use a Bonferroni correction given that we are using multiple statistical tests (7 times for each dataset), hence, for a difference to be considered statistically significant in this context the $p\text {-}value$ needs to be lower that 0.0071.

Some of these defects appeared in more than half of the images. This was the case of black dots, black spots and folds, which appeared in about $66\%$, $65\%$ and $61\%$ of the cases, respectively. Nonetheless, despite being less frequent, pen and watermarks, black edges, filaments and clear dyeing, appeared above $10\%$ of the times overall. Consequently, we could not dispense with them, or we would be dismissing a large portion of our data. Hence, our models had to deal with such features. On the other hand, the rest of defects were sufficiently rare in the training data that were unlikely to have a significant impact on the feature learning.

Table 1 shows the class imbalances present in these artefacts. Let us start with the MECC dataset, the largest differences across classes were found for black spots and pen marks, and they corresponded to a $9\%$ divergence. Consequently, we concluded that such imbalance was not high enough to constitute a significant concern. Since these defects were well represented in both classes $>20\%$, we expected that the algorithm would learn they are irrelevant for the classification task. When we looked at the TCGA case we found that the distribution of artefacts was more homogeneous and some artefacts did not appear at all in this dataset, such as black edges, clear dye, pen marks or broken glass. The largest imbalances were found for unusually darkened dye and tissue folds, both with a $7\%$ difference favouring the tumour class. Nonetheless, the relative difference is low and this is the test dataset, therefore, it would not introduce biases in the learning process.

**Table 1:** Ubiquity of the most frequent artefacts in both cohorts. The frequency of appearance is shown both per class (first and second columns) and in the total sample (third column). The last column corresponds to the $p\text {-}\text{value}$ from estimating the statistical significance of the difference in proportions across classes.

*MECC*	Tumour (%)	Normal (%)	Total (%)	*p*-value
Black dots	65	71	66	0.077
Black spots	67	58	65	0.0083
Folds	62	59	61	0.390
Pen marks	35	26	33	0.0078
Water marks	32	25	33	0.035
Black edges	29	23	31	0.063
Filaments	18	20	28	0.47
Clear dye	11	11	11	−
Black regions	8	9	9	−
Bubbles	11	4	8	−
Broken	5	2	5	−
Blurry	1	$<1$	1	−
TCGA				
Black dots	47	45	47	0.67
Black spots	73	74	74	0.81
Folds	70	63	68	0.11
Water marks	25	22	24	0.47
Filaments	35	36	35	0.83
Dark dye	23	16	20	0.078
Black regions	19	16	18	0.42
Bubbles	5	3	5	−
Blurry	4	3	3	−

### Image pre-processing

WSIs in full resolution were several gigabytes in size. Too large to be processed at once, image tiling or other downsampling techniques were needed. As is common practice, we downsampled the image area by tessellating the WSI and only taking the number of tiles that fitted into our GPU memory. However, this created the risk of relevant information loss, in particular of missing the tiles containing the tumour. This happened because our dataset lacked any prior annotations, i.e. there was no information on the distribution of tumour cells within the WSI. Therefore, sampling was performed randomly in order to probe the image evenly. This maximised the chances of sampling both healthy and cancerous tissue that might be present in the WSI. Although the risk of missing the cancer tissue altogether in a fraction of the cases was still present, this could be arbitrarily reduced by sampling the WSI thoroughly. As the GPU memory available was fixed, we decreased the resolution of the original WSI, which is another way of downsampling. In this way we were able to fully sample it via tiling. Of course, on one hand lowering the resolution may lead to the loss of the information contained in the finer details. On the other hand, if sufficient information is retained, this could be a powerful tool to make AI algorithms more efficient and their training more approachable, reducing computational costs significantly. Indeed, this is one of the things we wished to check in this work.

Taking all this into account, we proceed to summarise the data processing performed in this study: WSIs were square tessellated into a regular grid of non-overlapping tiles of dimensions $256 \times 256$ pixels. Tiles with above $75\%$ of their area corresponding to background were removed. Similarly, blurry regions and other defects were discarded using Canny edge detection from the OpenCV Python library. Those WSIs which produced fewer than $80/2^{R}$ valid tiles were also disregarded. *R* corresponds to the resolution level of the dataset, where $R=0$ and $R=3$ represent the highest and lowest resolution considered, respectively. Additionally, we used the Macenko normalisation method^17^ in order to minimise the bias produced by staining variations. All tiles were normalised using a reference image of matching resolution. In order to reduce the risk of overfitting we used data augmentation. The following set of transformations were randomly applied to the tiles: 90-degree rotations, vertical and horizontal flips and small ($\le 5\%$) variations in the brightness, saturation and contrast of the images. Finally, pixel values were rescaled from unsigned integers of 8 bits $\in [0, 255]$ to float 32 $\in [0, 1]$ and standardised by subtracting the mean and dividing by the standard deviation of the training dataset channel-wise. The means were (0.8566, 0.7857, 0.8633) and the standard deviations (0.1485, 0.1874, 0.1421). This has been shown to improve the convergence properties and accelerate the training process^18^.

Before any training had taken place, the dataset was split into training and test sets, with the proportions $90\%$ and $10\%$, respectively. We used cross-validation several times throughout this work, which means that the validation dataset was a variable subset of the training data. Finally, only after the preprocessing described before, the images were fed to the models.

### Multiple instance learning (MIL) classification

As mentioned before, medical images in high-resolution tend to take up a large amount of computational memory. Consequently, tessellating is inevitable in order handle them. However, labels often correspond to the entire WSI, with no spatial information. Which means, when the image is divided there is one label for a set of tiles of the WSI. And, in general, not every tile contains the relevant information for the inherited label, which may lead to noise and other issues while training.

MIL^19,20^ is an ideal approach to tackle this type of issue^21^. In contrast with classical classification, where there is one label, $y_n$, per instance, $x_n$, MIL works with one shared label, *Y*, for a bag of instances $X=\{x_1, x_2, \dots , x_N\}$. Where *N* denotes the size of the bag. In a binary classification scenario, the labels take the values $y_i \in \{0, 1\}$. Hence, the main assumption of MIL is that if a bag has $Y=1$ there must be at least one instance in it whose $y_n=1$. In contrast, if $Y=0$ all instances in the bag are non-informative. This can be summarised as follows:

1 $$\begin{aligned} Y = {\left\{ \begin{array}{ll} 0& \text {iff } \sum \limits _{i=1}^N y_i = 0, \\ 1& \text {otherwise} \end{array}\right. } \end{aligned}$$

The bag labels follow a Bernoulli distribution with $P(X)\in [0, 1]$, where *P* is the probability of $Y=1$ for a given bag of instances *X*.

Lastly, the MIL formalism requires a pooling function. The pooling projects the bag into a feature space whose dimensionality is independent of the number of instances per bag. Such operator must be permutation invariant since we assume no ordering or dependency between the instances. This low-dimensional representation is then processed by the classifier network. Some popular examples of pooling functions are the mean or the maximum value among all the instances. We present our choice of pooling function in the coming section.

Summarising, when a WSI is divided into tiles and *N* of them are sampled, this is equivalent to working with a bag of *N* instances. Each bag of tiles inherits the parent label of the WSI. This makes MIL very useful for our purpose. This also allows us to perform predictions at the WSI or patient level, rather than making one prediction per instance. Consequently, this method is more robust as it uses more effectively the full information contained in each WSI.

### Tumour location and the attention mechanism

We implemented attention as the MIL pooling function, i.e. it combines the information of all the features extracted from all the instances in a bag. In the past, this method had proven to be one of the best aggregation functions in the context of DL^22,23^. Attention is the primary component of “transformers”, an architecture that efficiently extracts contextual information, making it widely utilised across various fields^24^.

The mechanism of attention is a weighted mean where the weights are learned during the training process. It can be implemented as an additional DL model which is coupled to the main MIL architecture. It takes in the features extracted from each instance and processes them to produce one attention score ($p_{att}$) per instance. Such scores are proportional to how relevant or informative the instances are. These scores need to be transformed into weights ($w_{att}$) via the softmax operation, so they fulfil the condition $\sum _i^N w_{att,\;i} = 1$. Weights are then multiplied by the feature vector of the corresponding instance and all these products are added up together. This resulting feature vector, which summarises the entire WSI, is passed on to the classifier network. As previously mentioned, informative instances were assigned higher weights. This made the classification task easier and saved computational power. Furthermore, these attention scores could be pulled out from the model in order to identify those instances which were more relevant for the classification task. This has previously been done by^23^ as a way to pinpoint the tumour regions within the original WSI.

Even if we accomplish a model that accurately detects CRC, tumour tissue identification is a useful add-on. It may help pathologists as they visually inspect WSIs but, most importantly, provides insight into what the AI is doing. Often AI algorithms are seen as black boxes, meaning that it is really hard or impossible to disentangle how such algorithms make their “choices”. This is problematic because there is the risk that a DL model may learn wrong patterns, e.g. through overfitting or due to biases in the data, and therefore fail to generalise to new data. This is particularly concerning in safety-critical tasks such as cancer diagnosis. The attention mechanism^25,26^ allows us to identify the relevant tiles used to classify the WSI. Highlighting the parts of the image that the model was considering as crucial or more informative provided us with direct insight into the criteria that the AI was following. Let us consider once again the hypothetical case mentioned above in which a large fraction of the tumour samples has pen marks in the training dataset, originated from previous analysis of the same WSIs. Such bias is likely to be picked up by the model and quickly learn to identify artificial marks in the images. Such behaviour would be hard to notice without a way to look inside the black box. Nonetheless, attention would show that the model is giving high importance to pen mark-containing tiles while disregarding the actual tumour tissue. This makes attention a priceless ally to combine with some DL algorithms.

### Model architecture

**Fig. 3:**
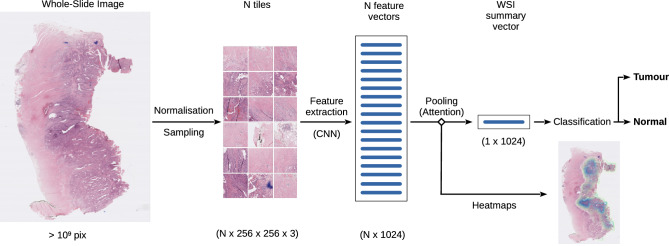
Schematics of the workflow through the DL pipeline, from the raw WSI to the classification outcome. The workflow steps are the following: (1) colour normalisation, tessellation and sampling of the image (2) CNN feature extraction (3) use of attention to aggregate all the feature vectors to produce one summary vector for the entire WSI (4a) DL classifier to produce the outputs of the model (4b) tumour heatmap generation using the attention scores.

Our approach consisted of an end-to-end DL pipeline which predicts at the WSI level, which is depicted in the diagram from Fig. 3. We adopted the widely-used feature extraction-then-classification structure. Feature extraction was carried out by a ResNet50 CNN with pre-trained weights on the ImageNet^27^ dataset ($\gtrsim 15$ million images). The CNN took bags of *N* instances each. Therefore, our feature extractor took $N\times 256\times 256\times 3$ stacks of images and returned arrays of vectors with $N\times 1024\times 1$ features. These were then passed through a single-headed attention module, which consisted of a fully-connected network with $1024\times 32$ and $32\times 1$ connections and $\tanh$ as activation function. The attention scores, one per instance, were passed through a softmax activation function to compute weights which added up to one. These were then multiplied by their corresponding feature vector and added so that the output had dimensions $1024\times 1$, which was independent on the bag size *N*. Finally, the classifier processed these features. The classifier was a fully-connected neural network with $1024\times 128$ and $128\times 2$ connections and rectified linear unit (ReLU) activation functions in between.

The model parameters quoted above were tuned using a randomised hyperparameter search. We used five-fold cross-validation on the training data for this part. Therefore, each configuration was trained five times allowing us to measure its robustness by computing the mean and standard deviation of losses and metrics. After this, the hyperparameter configuration that minimised the validation loss function was selected. When several models achieved equivalent performances, preference was given to the one with fewer parameters.

For the convolutional backbone of our model we chose the family of the residual networks (ResNet)^28^. ResNet was one of the first architectures to efficiently solve the vanishing gradients problem. Their solution was to introduce the so-called “identity shortcut connection”, which connects the outputs of different layers skipping the layers in between, forming a residual block. ResNets are constructed via stacking these residual blocks, therefore, there are many options of varying depth. We selected ResNet34 and ResNet50 because the former is the deepest ResNet architecture with an output vector of 512 features, while the latter is the shallowest one to produce 1024 features. Hence, in this way we test two feature vector sizes.

The hyperparameters values that were tested are the following:

- WSI resolution level (R): (0, 1, 2, 3)
- Learning rate: logarithmically sampled $\in \left( 10^{-5}, 10^{-1}\right)$
- Backbone: ResNet34 or ResNet50
- Bag size: $$2^n ~\forall ~ n \in [5, 8]$$
- Batch size: $$2n ~\forall ~ n \in [1, 12]$$
- Neurons in the classification layer: {64, 128}
- Number of classification layers: {1, 2}
- Neurons in the attention layer: {32, 64, 128}
- Number of attention layers: {1, 2}
- Number of epochs: {20, 60, 100}

### Training and validation

The training process involved a defined number of passes (epochs) of the training dataset through the model. In a single epoch, all WSIs in the training dataset were sampled. The sampling process was stochastic, i.e. *N* tiles were randomly selected from each WSI during each iteration. Therefore, sampling varied slightly in each epoch, which is beneficial for better exploiting the dataset, particularly when the bag size was smaller than the total number of tiles per WSI. Additionally, it served to mitigate the risk of overfitting as it has the same effect as data augmentation.

The training parameters used were the following: cross-entropy loss function and an adaptive moment estimation (ADAM) optimiser with an initial learning rate of 0.0001. The total number of epochs was set to 60. The criteria used to choose these and other hyperparameters is described in Sect. 2.6.

Both datasets used exhibited moderate imbalance, with cancer samples outnumbering normal ones. In the MECC dataset, $80\%$ of the WSIs were tumour samples, while the remaining $20\%$ were normal ones. TCGA showed a slightly higher imbalanced, with $91\%$ being tumour samples. Although this imbalance was not severe, we selected robust metrics against data imbalance. I.e. F1-score, area under the receiver operating characteristic curve (AUROC) and Matthews correlation coefficient (MCC). MCC measures the degree of association of two binary variables. To this date it is one of the most informative and robust measures of classification accuracy^29^. Its formula in terms of the number of true positive (*TP*), true negative (*TN*), false positive (*FP*) and false negative (*FN*) cases is as follows:

2 $$\begin{aligned} MCC = \frac{TP\times TN - FP\times FN}{\sqrt{(TP + FP)(TP + FN)(TN + FP)(TN + FN)}} \end{aligned}$$

Precision and recall were also tracked alongside the aforementioned metrics.

Finally, this work was implemented in a Python 3.9 environment, using the Pytorch 1.10.1 DL library to build and train our models. In terms of hardware, we used two NVIDIA GPUs, Geforce RTX 3090, with 24 GB each.

### Experimental design and statistical analysis

Since our main objective was to prove the true generalising capability of our model, we kept the TCGA data separate for final model testing. Therefore, the model building steps were carried out using the MECC dataset. From this dataset we also saved $10\%$ of the images for testing. The remaining $90\%$ was used for model selection, training and validation.

Our experimental design follows the subsequent steps: First we used hyperparameter tuning to find the optimal configurations and best performing models for each image resolution (more details in Sect. 2.6). After identifying the best configuration, the corresponding model was trained repeatedly (100 times) on the MECC images, leaving out the test sample. Train and validation data were selected at random, with the fixed proportion 90/10, in each training process. The result of each training was then tested on the datasets reserved for this purpose. Eventually we determined the distribution of metric values by calculating the mean and standard deviation of all realisations. In Sect. 3.4 we conducted two ablation studies to independently assess the contributions of attention pooling and colour normalisation to overall model performance. Finally, we analysed the outputs of the attention mechanism in order to generate tumour heatmaps with this final model, both in the MECC test and TCGA data in Sect. 3.5.

## Results

In this section we present the main outcomes of our work, including the best DL models at each WSI resolution level and compare them. Furthermore, we analyse the cost-efficiency in terms of time and resources. We then tested the classification accuracy of the best model both on the MECC and TCGA datasets. Finally, we explored the ability of this model to produce accurate tumour heatmaps using the attention mechanism scores.

### Model performance as a function of resolution

The hyperparameters that correlated more strongly with the image resolution were the bag size and the batch size. The rest of them were unchanged across all four resolution levels. Furthermore, moderate variations on the values of those hyperparameters had negligible impact on the performance of the model. Therefore, the common hyperparameter values were a learning rate of $10^{-4}$ with 60 epochs of training; a ResNet50 as convolutional backbone of the model; the attention network with just one hidden layer with 32 neurons and the classifier also with one hidden layer with 128 neurons. The hyperparameters that showed dependence with resolution, i.e. batch and bag size, were presented in Table 2 for clarity.

**Table 2:** Optimal bag size and batch size as a function of WSI resolution level.

R	0	1	2	3
Bag size	256	64	64	32
Batch size	4	12	12	24

In Table 3 we show the averaged results across a 5-fold cross-validation run for each model. The macro average of the main metrics is presented: precision, recall, F1 score, AUROC and MCC. It shows that we obtained good results in all experiments conducted with the MECC data.

**Fig. 4:**
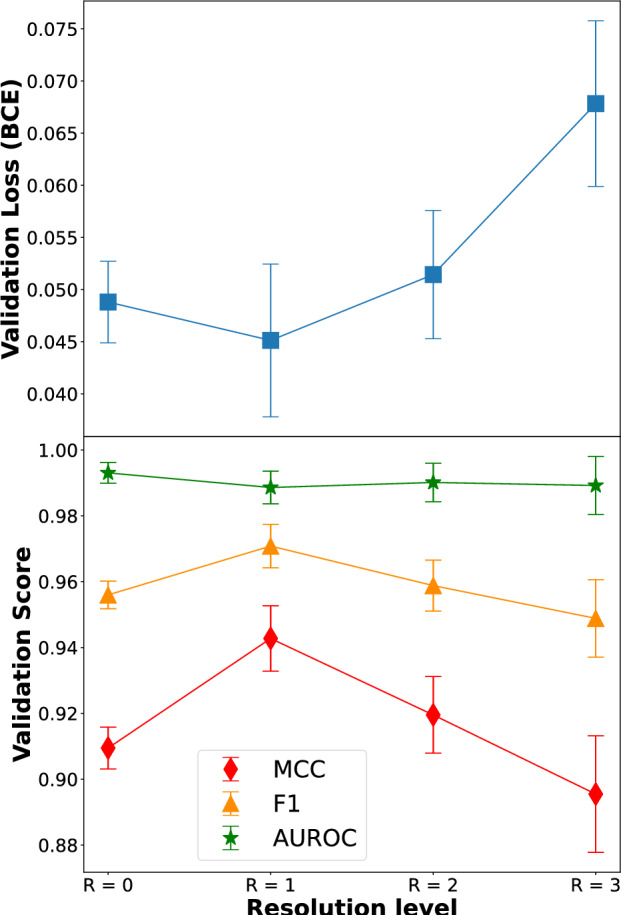
Performance as a function of resolution level. On the top plot the validation loss (BCE) is represented, on the bottom one we see the main metrics: MCC, F1 score and AUROC; evaluated on the validation dataset.

The best performance corresponded to the resolution level $R=1$ ($4.0$ μm/pix), with $\text {MCC}=0.943$ and $F1_{Macro}=0.971$. Such values are excellent and prove our model to be efficient at classifying WSIs of colorectal tissue. From Table 3 we also see that $\text {recall}_{Macro}=0.977$, but if we only looked at the positive class, we obtain a recall of 0.990, from which we infer a false negative rate (FNR) of $1\%$. While the false positive rate (FPR) was $19.0\%$. On the other hand, despite presenting the worst performance metrics, $R=3$ ($16.0$ µm/pix) achieved values as high as $\text {MCC}=0.895$ and $F1_{Macro}=0.949$, which still corresponded to a highly accurate model. The tumour recall score was 0.967 which meant that the FNR was $<4\%$. In summary, we found that the same model performed best with images of $4.0$ µm/pix resolution, followed by $2.0$ µm/pix, $8.0$ µm/pix and $16.0$ µm/pix, in decreasing order. This can be seen in Fig. 4, where the validation losses are plotted alongside the metrics as a function of image resolution.

**Table 3:** Summary of the validation metrics obtained for the models at each resolution level. These values were calculated using 5-fold cross validation.

Resolution	Precision	Recall	F1-Score	AUROC	MCC
R=0	0.956	0.962	0.956	0.993	0.909
R=1	0.965	0.977	0.971	0.989	0.943
R=2	0.956	0.962	0.959	0.990	0.920
R=3	0.931	0.967	0.949	0.989	0.895

### Cost-efficiency analysis

We evaluated the computational cost of our approach in terms of training throughput, hardware utilisation, and cost per experiment. Training required 1.5–3 min per epoch, amounting to approximately 1.5–3 hours for 60 epochs, with both GPUs operating at 90–100% utilisation.

On-the-fly tile selection increased training time by approximately $30\%$ compared with pre-encoded approaches, but this choice was integral to our end-to-end design, which trains the convolutional encoder from scratch. Pre-encoding was therefore not feasible, and storing pre-augmented images would have required several hundred gigabytes of additional storage. Given the modest overhead, we considered the trade-off acceptable.

Moreover, on-the-fly augmentation ensures the model never encounters the same augmented image twice, providing virtually unlimited variation and improved generalisation. As augmentation is disabled during validation and inference, the additional cost is confined to training.

Overall, our approach achieves a favourable balance between generalisation and computational efficiency while optimising storage use.

### Intra- and inter-cohort testing

**Fig. 5:**
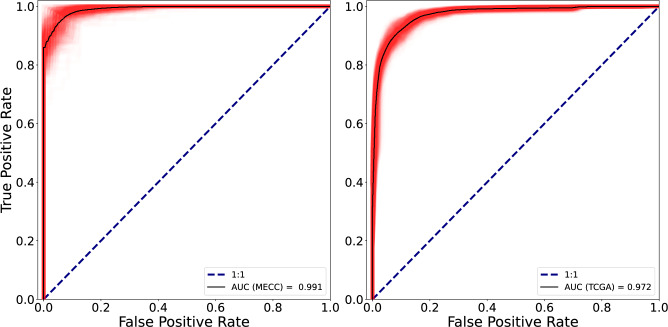
ROC curves of our model evaluated on the test datasets, the left one corresponds to the MECC and the right one to the TCGA data. In red we show the distribution of ROCs corresponding to different training of the model on the same dataset. The main sources of variation are the cross validation runs and the stochasticity of the tile selection procedure, so that the test data are never identical between realisations. The black solid line corresponds to the mean ROC curve.

In this section we evaluate the performance of our model on previously unseen data. First on the fraction of the MECC data that had been kept separate and then on the TCGA images. The results are summarised on Table 4, where the values of the loss function, precision, recall, MCC, F1 score and AUROC are displayed. Additionally, the ROC curves of the test datasets are plotted in Fig. 5.

First and foremost, we observed that performance was not significantly different between the internal and external test datasets, with respective values of the BCE loss of 0.112 and 0.134. This indicated that or model generalised well, which also reflected on the high values of the metrics. For instance, MCC was always above 0.7 which showed high degree of correlation between predictions and ground truth values. Just for the purpose of comparison with other studies we give the accuracy value, despite it not being a good measure since we work with imbalanced data. The accuracy values we obtained are $0.972 \pm 0.010$ for the MECC data and $0.957 \pm 0.006$ for the TCGA.

Furthermore, we found that our model was robust, which was supported by the narrow confidence intervals ($\alpha = 0.05$). This means that across 100 training runs with randomised initialisation, rolling training and validation sets and the stochastic contribution from image sampling and augmentation, the model converged to a similar and stable solution. This can also be seen in Fig. 5 where the distribution of ROC curves (red region) tightly clustered around the mean value (black solid line), with no strong outliers.

**Table 4:** Loss and metric values evaluated on the test datasets. The top three rows correspond to intra-cohort testing on the MECC dataset, while the the bottom three correspond to inter-cohort testing on the TCGA dataset. Within each group, the first row presents the results using the full model with attention, and the second and third rows report results from the ablation study, where attention is replaced with average and max pooling, respectively. 95% confidence intervals were estimated via repeated training with changing initialisation and training/validation sets.

	Pooling	BCE	Precision	Recall	F1-Score	AUROC	MCC
	Attention	$0.112 \pm 0.012$	$0.954 \pm 0.008$	$0.961 \pm 0.008$	$0.957 \pm 0.007$	$0.991 \pm 0.006$	$0.915 \pm 0.014$
MECC	Average	$0.237 \pm 0.022$	$0.817 \pm 0.014$	$0.981 \pm 0.010$	$0.900 \pm 0.009$	$0.920 \pm 0.008$	$0.828 \pm 0.016$
	Max	$0.269 \pm 0.020$	$0.728 \pm 0.012$	$0.905 \pm 0.011$	$0.810 \pm 0.007$	$0.945 \pm 0.010$	$0.699 \pm 0.017$
	Attention	$0.134 \pm 0.009$	$0.922 \pm 0.017$	$0.822 \pm 0.021$	$0.863 \pm 0.018$	$0.972 \pm 0.007$	$0.737 \pm 0.033$
TCGA	Average	$0.300 \pm 0.013$	$0.784 \pm 0.013$	$0.855 \pm 0.023$	$0.826 \pm 0.013$	$0.901 \pm 0.011$	$0.687 \pm 0.027$
	Max	$0.317 \pm 0.017$	$0.737 \pm 0.011$	$0.839 \pm 0.019$	$0.794 \pm 0.013$	$0.907 \pm 0.012$	$0.599 \pm 0.025$

### Ablation studies

Two ablation studies were utilised to analyse the measure the performance gains obtained by implementing attention and pooling the colour normalisation method.

To evaluate the contribution of attention pooling, we compared its performance against average and max pooling through an ablation study, summarised in Table 4. The results demonstrate that attention pooling consistently outperforms fixed pooling strategies across both intra-cohort (MECC) and inter-cohort (TCGA) test settings.

We observe that attention pooling led to substantial improvements, particularly in precision and MCC, with gains up to 22.6 and 21.6% respectively on the MECC dataset, and 18.5 and 13.8% on the TCGA. Additionally, BCE loss was reduced more than 50% in both cohorts. Although recall remained high across all pooling methods, attention pooling achieved better balance between precision and recall, resulting in superior F1 score. Moreover, attention pooling yielded higher AUROC values, suggesting improved overall discriminating power. Finally, we also note that attention pooling mitigates performance drops in the external test, suggesting better generalisation capability than classical methods.

The second ablation study examined the effect of removing Macenko colour normalisation from the preprocessing pipeline, therefore using the unprocessed tiles directly extracted from the raw WSIs. The results indicate that colour normalisation is a critical component of the pipeline. When omitted, the model fails to generalise, returning AUROC values consistently around 0.5, equivalent to random guessing. This highlights the importance of colour normalisation for DL computer vision tasks.

### Tumour heatmaps

We shared a number of heatmaps generated by the model with trained pathologists, so they could judge the ability its ability to identify tumour regions. We concluded that the accuracy of the heatmaps is very high. Given that computing metrics for this part was unfeasible, here we show a collection of examples to help visualise and interpret our results.

**Fig. 6:**
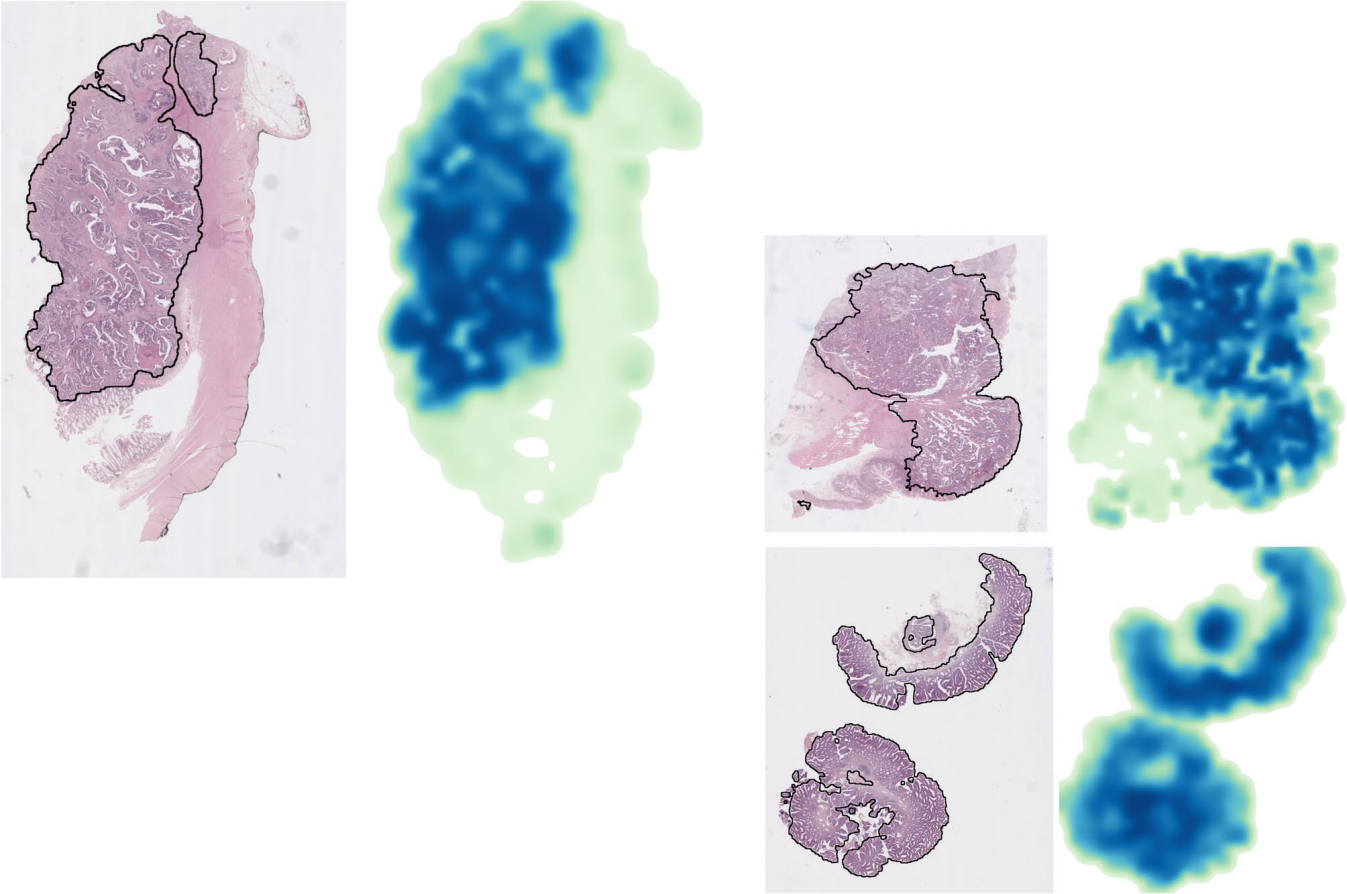
Three examples of tumour tissue location, each one is composed by a pair of images: the original WSI (on the left) with a manual delineation of the tumour tissue shown as a black continuous line, and the corresponding attention probability heatmap (on the right). Darker blue regions imply high probability of cancer, in contrast with the light green ones, which overlap with normal tissue.

Attention probability heatmaps were produced via creating a mosaic merging tiles with the value of the attention score. Several predictions were made in order to cover the area of the WSI and overlapping between tiles was allowed in order to increase the spatial resolution of the heatmaps. Where more than one prediction overlapped the mean of the attention scores was taken. Finally, the resulting heatmap was smoothed with a Gaussian filter in order to remove artificially induced pixelated appearance of the outcome. Such heatmaps were presented in Fig. 6, where they are displayed next to their corresponding WSI. The darker blue regions on the heatmaps correspond to the highly informative pixels, i.e. those containing tumour tissue. These examples show a good correlation between the patches of tumour tissue (mostly tumour mucosa) and the higher attention weights, while healthy mucosa, muscle and adipose tissue obtained low attention scores.

**Fig. 7:**
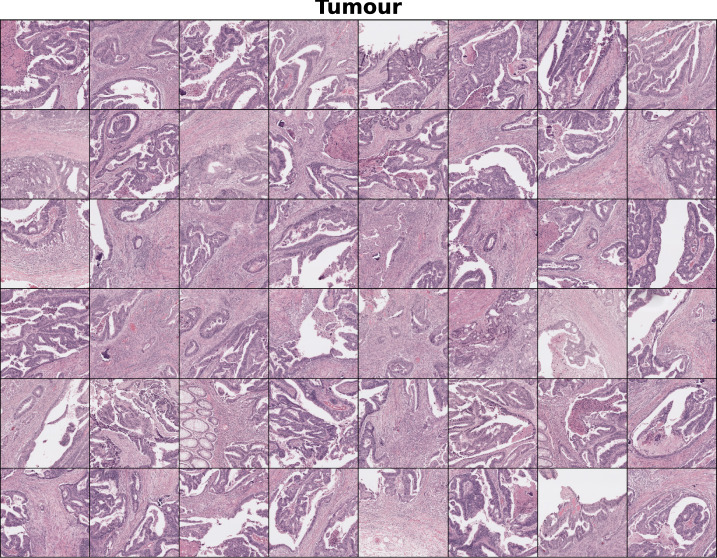
Collection of tumour instances which were selected for fulfilling $p_{att}$> 0.6. We appreciate unstructured colorectal mucosa and heavily dyed tissue, probably due to the abnormal growth of nuclei in tumour cells.

**Fig. 8:**
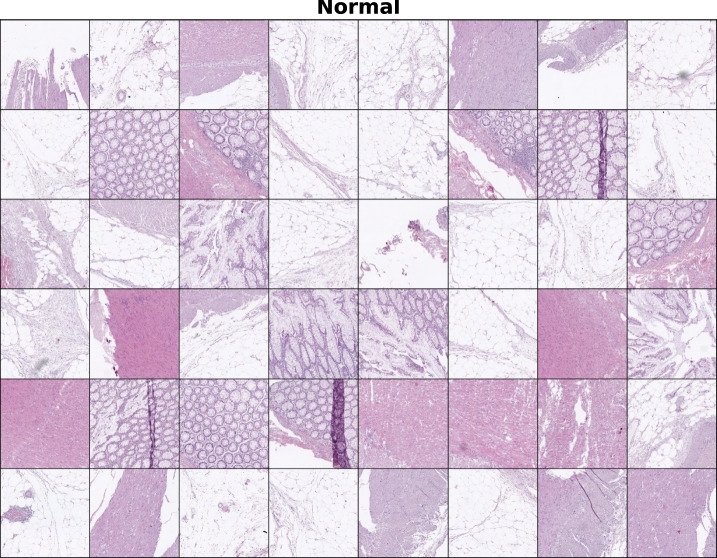
Collection of normal instances which were selected for fulfilling $p_{att}$< 0.4. In contrast with the tumour ones, in this case instances present different features such as regular, structured mucosa and other types of tissue, such as muscle and adipose tissue.

Additionally, we present a collection of $256\times 256$ pix tiles belonging to either class. In Fig. 7 we display a grid containing 48 tumour tiles, which were selected with the criterion $p_{att}>0.6$. In those we observe the presence of altered mucosae and heavily stained tissue, consequence of the enlarged nuclei typical of cancer cells. In contrast, in Fig. 8 where we find only healthy tiles ($p_{att}<0.4$), we observe predominantly normal mucosa as well as muscle tissue.

As expected, there were also some errors and strange behaviours. For example, we found a number of normal WSIs which were correctly classified, however, some of their tiles were assigned high attention scores. We discuss these and some other ambiguous cases in Sect. 4.2.

## Discussion

### Resolution versus sampling

WSIs in original resolution ($0.25$ µm/pix) were in the scale of the giga-pixel. Such images can be divided into around $10^5$ $256\times 256$ tiles. Whilst these retain all the spatial information, they are hard to handle since large bag sizes are required to sample a significantly large area of the image. Many existing studies only used one resolution level, typically 0.25 or $0.5$ µm/pix^30–32^. Such studies require important computational resources, usually powerful GPUs. These resources are expensive and may not always be available. We propose adapting the image resolution so that this computational power issue is mitigated while performance is kept within a reasonable threshold.

For example, a bag size of 100 instances covers only about $0.1\%$ of the native area of a WSI. So that it is impossible to probe the tumour regions sufficiently. At the maximum resolution of this study ($2$ µm/pix) one WSI gave rise to of the order of $10^3$ $256\times 256$ tiles. Hence, a bag size of 100 instances covered $10\%$ of the WSI, which meant a significant improvement but is still low. In the case of $4$ µm/pix the number of tiles was reduced to $\sim \!\!300$, which meant that our bag size sampled about a third of the total image. The following steps were $8$ µm/pix, with $\lesssim 100$ instances per WSI, and $16$ µm/pix, with just tens of them. In the last two cases we were able to sample the $100\%$ of the image. Nevertheless, the negative impact of lowering the resolution was more likely to dominate in the last cases. The lower the resolution the higher the risk of suffering critical information loss, as the finer details get blurred out or completely erased.

The aforementioned compromise between image resolution and sampling is likely to explain our findings. I.e. the best performance was achieved with an intermediate resolution rather than with either of the extremes. It is worth mentioning that, even though the described dependence with resolution was found, the effect was not very strong. The DL models performed well in all resolution levels tested. This indicates that lowering the resolution is a viable option which incidentally reduces the computational costs while maintaining a good performance.

### Insights from attention

In Sect. 3.5 we saw that attention did a good job at identifying cancerous tissue on images classified as tumour. In this section we cherry-pick a set of ambiguous examples to discuss them in detail and gain a deeper understanding of our model and its limitations.

**Normal tissue with high attention score**

Let us examine some instances that were assigned high attention scores even though their WSI was correctly classified as normal.

**Fig. 9:**
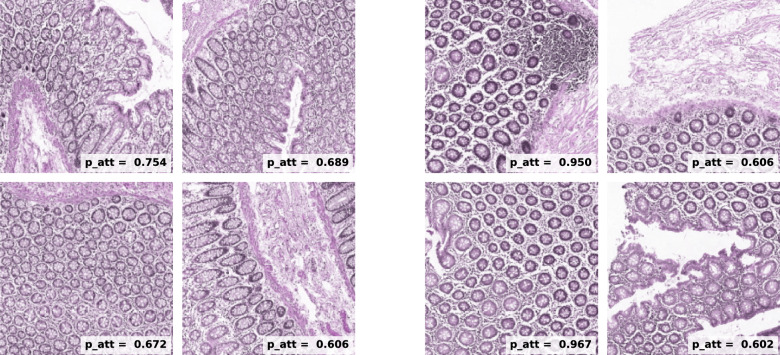
Examples of correctly classified normal tiles, each with its corresponding attention score on the bottom right corner. All eight cases were selected because the have been assigned high attention scores (> 0.6). It is to be noted that the four tiles on the right-hand-side of the panel present inflammatory signatures demonstrated by abundant lymphoid aggregates.

In the first example of Fig. 9 we see a section of normal colorectal mucosa which had, on the contrary, been assigned high attention scores (all $p_{att}$ are well above 0.5). Note that in this case the mucosa preserves its structure and there is no evidence indicating abnormality.

The attention mechanism assigns higher scores to those instances which are informative for the classification task. This means that attention scores are conditioned by the predicted class, i.e. if a slide is negative, we expect patches conveying negative evidence to be assigned high attention scores. Ideally, the presence of tumour tissue is the only informative characteristic, in contrast with normal tissue which may appear both in normal and tumour WSIs. Nonetheless, in our dataset tumour WSIs are dominated by tumour tissue, i.e. almost all mucosae are affected by cancer. This led the model to associate normal mucosa with cancer-free WSIs, considering it informative for the normal category. Although this effect originated from a bias in the training data, given the good performance of algorithm we can conclude it does not negatively affect our results. Furthermore, we presume the ability of our model to take into account the context information counteracts this effect. As proof of this, the discussed example case was correctly classified despite the high $p_{att}$ values obtained.

**Fig. 10:**
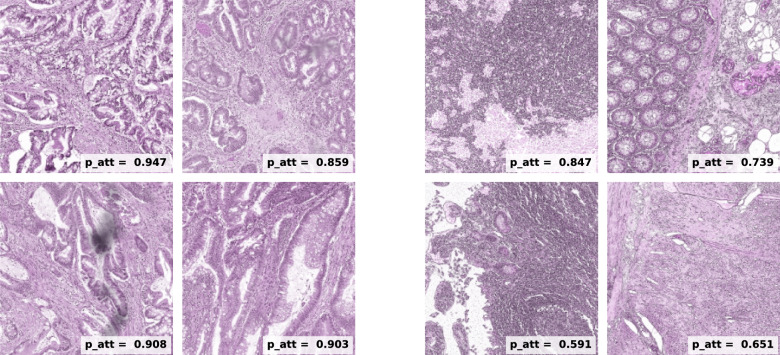
The tiles presented here correspond to discrepancies between the prediction and the ground truth. In particular, these cases correspond to normal WSIs which were classified as tumour by the model. However, the four tiles on the left-hand-side of the panel present clear evidence of cancer. On the other hand, the tiles on the right-hand-side present a strong inflammatory response.

In the second example of Fig. 9 we observe that the colon mucosa looks normal. Nonetheless, a high level of lymphoid infiltration and aggregates are found. It is possible that the algorithm is associating an inflammatory response with the tumour category. Although some patients develop a Crohn’s-like lymphoid reaction to CRC, such feature is not unique to CRC and can be associated with other conditions. Therefore, this could be problematic and a source of false positives (see another similar example in Fig. 10)

**Normal tissue incorrectly classified**

Let us now go through some cases in which the model misclassified normal WSIs as cancerous.

In Fig. 10, on the right-hand-side, we show another example of inflammatory response evident by the abundant infiltration of lymphocytes within the tissue. The possibility of Crohn’s-like reaction triggering false positives is a caveat to our method, nonetheless, it is not a major concern given that it produces false positives rather than false negatives. Therefore, it will not cause cancer cases to go undetected. This effect will possibly be mitigated with a larger dataset, and particularly with more WSIs in the normal category.

The last example we will analyse is found on the left-hand-side of Fig. 10. We see four instances containing colorectal mucosa which, in contrast with the previously shown, show a rather irregular pattern. This is an indication of tumour tissue and a pathologist confirmed it to be so. Therefore, our method was able to find a mislabelled WSI in the dataset. A fraction of these is always expected to be present due to human error. Therefore, it is a good sign that our model was able to classify it correctly.

### Comparison with advances in the field

In this section we put our study in context and compare it with similar ones as to evaluate the progress achieved in this field in recent years.

The study conducted by^33^ used a combination of low- and high-resolution versions of the WSIs in order to deal with WSI-level annotations. A low-resolution thumbnail of the WSI was fed to their model in order to account for the global information. Simultaneously, square tiles were cropped from the highest resolution image to use the cell-level information as well. A classifier was then trained only on normal tiles, so that the model could learn the features of normal images. So that the model was able to detect cancerous images according to the degree to which they differed from the normal ones. Finally, they used the probability of each tile of containing cancer to produce heatmaps similar to the ones we have shown in this work. The dataset they used was part of the TCGA, reaching an accuracy of $94.6\%$ on test data and $92.0\%$ on newly collected images.

Another similar study by^32^ used $>\!\!14680$ WSIs from $>\!\!9631$ patients, one of the largest samples to-date. They developed a patch aggregation strategy for weakly labelled images which achieved metrics as high as $AUROC=0.988$. Furthermore, they claim that on average the AI slightly outperformed the predictions by six pathologists ($AUROC_{\text {pathologist}} = 0.970$) that took part in their study.

Another study which used instance level classification is^30^, where they trained a CNN to identify 9 different types of tissue in histopathology WSIs. They achieved an overall accuracy of $99\%$ on the internal testing set and $94.3\%$ on the external one. However, this fully supervised approach required instance level labels or at least WSI spatial information.

In^34^ a similar model to ours, which they named clustering-constrained-attention multiple-instance learning (CLAM), was presented. Such method provides a WSI-level representation and classification based on MIL and attention, as well as automatic identification of sub-regions of high diagnostic value. The main advantage of our method is that it corresponds to an end-to-end approach, which is described by^34^ as potentially better for performance yet hard to accomplish due to data and computational constraints. WSI downsampling allowed us to work around the issue and obtain a highly data-efficient model. Besides, CLAM was trained on different datasets including kidney, lung and breast cancer and, in contrast with our approach, attempted subtyping these cancer samples. Despite the differences between studies it is worth the comparison due to the similarity in the algorithms involved.^34^ reached test AUCs in the range 0.953–0.991, which is comparable to ours. Nevertheless, it has been found to decrease slightly when applied to other types of cancer, e.g. Ghaffari-Laleh et al.^35^, with values between 0.671 and 0.795.

We could not close this section without analysing our work in the context of transformers. Although our algorithm may be regarded as a particular case of a transformer, which uses convolutional layers for encoding features and with single-headed attention, we keep such denomination for more complex models with multi-head attention and positional encoding, such as the model developed by^36^. In fact, their analysis allows them to take into account the spatial information between instances. Nevertheless, we see that our performance is similar to theirs.

Since the list of relevant studies is endless, we refer the reader to^37^ for a recent review on applications of DL to histopathological imaging for CRC diagnosis. Where a thorough comparison of existing studies and their outcomes is presented.

We see that our model reaches similar or higher performance than analogous state-of-the-art studies on DL for cancer diagnosis through histopathology images.

To finish, we comment on the main limitations of our model and potential improvements. The amount of data we used, although enough, is on the shorter side. Meaning that this study would benefit from a larger and more diverse dataset. For example, with images from different datasets which belong to different WSI cohorts, as well as a larger fraction of normal WSIs.

Currently our weakly annotated images carry the human biases and human errors with them. While using supervised learning the models will be limited by the skills that had the experts who labelled the data used for training. Although these models are more deterministic, getting rid of the subjective variations, and even find some hidden patterns undetected before, we should not expect these models to greatly improve human performance. It could be enlightening to apply self-supervised learning (SSL)^38,39^ to this problem. SSL is a subset of unsupervised learning and, therefore, has the potential to explore and exploit new features present in the images that are informative but escape human eye. On the other hand, this approach could be hard to implement in a context with such large images. Nonetheless, its implementation together with MIL could throw some interesting results worth pursuing.

## Conclusions

We have conducted a comprehensive study about the performance of a deep learning model that integrates MIL and attention mechanism for the classification of histopathological WSIs into normal and tumorous categories.

We have studied the performance of a DL model which combines MIL and attention in classifying histopathological WSIs into normal and tumorous. Furthermore, we have analysed the optimal image resolution within this context. Additionally, our model has been engineered to locate tumour tissue within the WSI. All these within a single end-to-end pipeline, easy to optimise and implement.

In culmination, tested our best-performing model on independent images, from both the same and an external cohort, to evaluate its generalisation power. Below we summarise our main conclusions:

1. Like similar studies, we find that DL is a suitable candidate to process and analyse medical images. In particular, MIL and the attention mechanism make a good association and reach high accuracy in the task of cancer detection through WSI classification.
2. WSI classification using DL does not seem to strongly rely on the resolution of the images. This allows us to lower the resolution without causing significant damage to the classification performance. Furthermore, given our computational constraints, we find an optimal working resolution of 4.0 μm/pix ($16\times$ lower than the highest available). In a regime where a compromise between efficient image sampling and fine detail losses is accomplished.
3. The attention mechanism allows us to have a glance inside the DL “black box” and therefore provides a valuable tool for explainability and interpretability of DL models. Furthermore, directly using the attention scores we can automatically identify the tumour regions within the large WSIs.
4. It is possible to train DL algorithms that are general enough to perform satisfactorily on separate cohorts from the one used for training. This has always been a major concern because different cohorts present intrinsic variations such as, the equipment that was used, the amount of dye, the amount of tissue present, among others.
5. We have found that DL complements pathologist skills well as they have different strengths and weaknesses. Furthermore, AI can greatly speed up the process of diagnosis as these algorithms can process several WSIs per minute. In addition to which they can tell the pathologist were to look beforehand, so they may not need to explore the entire WSI in detail. Additionally, the AI could automatically screen some highly reliable cases so the pathologist may not be required to scrutinise all the WSIs.

We also conclude that increasing the size of our dataset is likely to improve the results. Specially including images from different cohorts into the training dataset would potentially boost the generalisation power of the model. Similarly, some novel techniques such as SSL may also improve the outcomes of classification with weakly labelled data, which are often found in the field of medicine.

## Data Availability

The MECC dataset, used for training our model, is not publicly available due to restrictions that required IRB approval to use the data, but is available from SBG on reasonable request. The TCGA dataset analysed during the current study is available in the public repository (https://portal.gdc.cancer.gov/).

## References

[1] Sung, H. et al. Global cancer statistics 2020: Globocan estimates of incidence and mortality worldwide for 36 cancers in 185 countries. *CA Cancer J. Clin.* **71**, 209–249 (2021). 33538338 10.3322/caac.21660

[2] Kelly, M., Soles, R., Garcia, E. & Kundu, I. Job stress, burnout, work-life balance, well-being, and job satisfaction among pathology residents and fellows. *Am. J. Clin. Pathol.* **153**, 449–469. 10.1093/ajcp/aqaa013 (2020). 32080717 10.1093/ajcp/aqaa013

[3] Aeffner, F. et al. Introduction to digital image analysis in whole-slide imaging: A white paper from the digital pathology association. *J. Pathol. Inf.* **10**, 9. 10.4103/jpi.jpi_82_18 (2019). 10.4103/jpi.jpi_82_18 PMC6437786 30984469

[4] Öztürk, Ş & Akdemir, B. Cell-type based semantic segmentation of histopathological images using deep convolutional neural networks. *Int. J. Imaging Syst. Technol.* **29**, 234–246 (2019).

[5] Huang, S., Yang, J., Fong, S. & Zhao, Q. Artificial intelligence in cancer diagnosis and prognosis: Opportunities and challenges. *Cancer Lett.* **471**, 61–71. 10.1016/j.canlet.2019.12.007 (2020). 31830558 10.1016/j.canlet.2019.12.007

[6] Thakur, N., Yoon, H. & Chong, Y. Current trends of artificial intelligence for colorectal cancer pathology image analysis: A systematic review. *Cancers*10.3390/cancers12071884 (2020). 32668721 10.3390/cancers12071884 PMC7408874

[7] Hua, K.-L., Hsu, C.-H., Hidayati, S. C., Cheng, W.-H. & Chen, Y.-J. Computer-aided classification of lung nodules on computed tomography images via deep learning technique. *OncoTargets Ther.* **8** (2015). 10.2147/OTT.S80733 PMC4531007 26346558

[8] Coudray, N. et al. Classification and mutation prediction from non-small cell lung cancer histopathology images using deep learning. *Nat. Med.* **24**, 1559–1567 (2018). 30224757 10.1038/s41591-018-0177-5 PMC9847512

[9] Yu, L., Chen, H., Dou, Q., Qin, J. & Heng, P.-A. Automated melanoma recognition in dermoscopy images via very deep residual networks. *IEEE Trans. Med. Imaging* **36**, 994–1004 (2016). 28026754 10.1109/TMI.2016.2642839

[10] Veta, M. et al. Assessment of algorithms for mitosis detection in breast cancer histopathology images. *Med. Image Anal.* **20**, 237–248. 10.1016/j.media.2014.11.010 (2015). 25547073 10.1016/j.media.2014.11.010

[11] Kashyap, R. Evolution of histopathological breast cancer images classification using stochasticdilated residual ghost model. *Turk. J. Electr. Eng. Comput. Sci.* **29**, 2758–2779 (2021).

[12] Campanella, G. et al. Clinical-grade computational pathology using weakly supervised deep learning on whole slide images. *Nat. Med.* **25**, 1301–1309 (2019). 31308507 10.1038/s41591-019-0508-1 PMC7418463

[13] Bulten, W. et al. Automated deep-learning system for Gleason grading of prostate cancer using biopsies: A diagnostic study. *Lancet Oncol.* **21**, 233–241 (2020). 31926805 10.1016/S1470-2045(19)30739-9

[14] Babu, T., Singh, T., Gupta, D. & Hameed, S. Optimized cancer detection on various magnified histopathological colon imagesbased on dwt features and FCM clustering. *Turk. J. Electr. Eng. Comput. Sci.* **30**, 1–17 (2022).

[15] Roy, S., Kumar-Jain, A., Lal, S. & Kini, J. A study about color normalization methods for histopathology images. *Micron* **114**, 42–61. 10.1016/j.micron.2018.07.005 (2018). 30096632 10.1016/j.micron.2018.07.005

[16] Kothari, S. et al. Removing batch effects from histopathological images for enhanced cancer diagnosis. *IEEE J. Biomed. Health Inform.* **18**, 765–772 (2013). 10.1109/JBHI.2013.2276766 PMC5003052 24808220

[17] Macenko, M. et al. A method for normalizing histology slides for quantitative analysis. In *2009 IEEE International Symposium on Biomedical Imaging: From Nano to Macro*, 1107–1110. 10.1109/ISBI.2009.5193250 (2009).

[18] Ioffe, S. & Szegedy, C. Batch normalization: Accelerating deep network training by reducing internal covariate shift. In *International conference on machine learning*, 448–456 (PMLR, 2015).

[19] Maron, O. & Lozano-Pérez, T. A framework for multiple-instance learning. In Jordan, M., Kearns, M. & Solla, S. (eds.) *Advances in Neural Information Processing Systems*, vol. 10 (MIT Press, 1997).

[20] Dietterich, T. G., Lathrop, R. H. & Lozano-Pérez, T. Solving the multiple instance problem with axis-parallel rectangles. *Artif. Intell.* **89**, 31–71. 10.1016/S0004-3702(96)00034-3 (1997).

[21] Quellec, G., Cazuguel, G., Cochener, B. & Lamard, M. Multiple-instance learning for medical image and video analysis. *IEEE Rev. Biomed. Eng.* **10**, 213–234. 10.1109/RBME.2017.2651164 (2017). 28092576 10.1109/RBME.2017.2651164

[22] Vaswani, A. et al. Attention is all you need. *Advances in neural information processing systems* **30** (2017).

[23] Ilse, M., Tomczak, J. & Welling, M. Attention-based deep multiple instance learning. In *International Conference on Machine Learning*, 2127–2136 (PMLR, 2018).

[24] Dosovitskiy, A. et al. An image is worth 16x16 words: Transformers for image recognition at scale. *arXiv preprint*arXiv:2010.11929 (2020).

[25] Bahdanau, D., Cho, K. & Bengio, Y. Neural machine translation by jointly learning to align and translate. *arXiv preprint*arXiv:1409.0473 (2014).

[26] Raffel, C. & Ellis, D. P. Feed-forward networks with attention can solve some long-term memory problems. *arXiv preprint* arXiv:1512.08756 (2015).

[27] Deng, J. et al. Imagenet: A large-scale hierarchical image database. In *2009 IEEE Conference on Computer Vision and Pattern Recognition*, 248–255 (IEEE, 2009).

[28] He, K., Zhang, X., Ren, S. & Sun, J. Deep residual learning for image recognition. In *2016 IEEE Conference on Computer Vision and Pattern Recognition (CVPR)*, 770–778, 10.1109/CVPR.2016.90 (2016).

[29] Chicco, D., Warrens, M. J. & Jurman, G. The Matthews correlation coefficient (mcc) is more informative than Cohen’s kappa and brier score in binary classification assessment. *IEEE Access* **9**, 78368–78381. 10.1109/ACCESS.2021.3084050 (2021).

[30] Kather, J. N. et al. Predicting survival from colorectal cancer histology slides using deep learning: A retrospective multicenter study. *PLoS Med.* **16**, 1–22. 10.1371/journal.pmed.1002730 (2019). 10.1371/journal.pmed.1002730 PMC6345440 30677016

[31] Kather, J. N. et al. Deep learning can predict microsatellite instability directly from histology in gastrointestinal cancer. *Nat. Med.* **25**, 1054–1056 (2019). 31160815 10.1038/s41591-019-0462-y PMC7423299

[32] Wang, K.-S. et al. Accurate diagnosis of colorectal cancer based on histopathology images using artificial intelligence. *BMC Med.* **19**, 1–12 (2021). 33752648 10.1186/s12916-021-01942-5 PMC7986569

[33] Zhou, C. et al. Histopathology classification and localization of colorectal cancer using global labels by weakly supervised deep learning. *Comput. Med. Imaging Graph.* **88**, 101861. 10.1016/j.compmedimag.2021.101861 (2021). 33497891 10.1016/j.compmedimag.2021.101861

[34] Lu, M. Y. et al. Data-efficient and weakly supervised computational pathology on whole-slide images. *Nat. Biomed. Eng.* **5**, 555–570 (2021). 33649564 10.1038/s41551-020-00682-w PMC8711640

[35] Ghaffari Laleh, N. et al. Benchmarking weakly-supervised deep learning pipelines for whole slide classification in computational pathology. *Med. Image Anal.* **79**, 102474. 10.1016/j.media.2022.102474 (2022). 35588568 10.1016/j.media.2022.102474

[36] Shao, Z. et al. Transmil: Transformer based correlated multiple instance learning for whole slide image classification. *Adv. Neural. Inf. Process. Syst.* **34**, 2136–2147 (2021).

[37] Davri, A. et al. Deep learning on histopathological images for colorectal cancer diagnosis: A systematic review. *Diagnostics*10.3390/diagnostics12040837 (2022). 35453885 10.3390/diagnostics12040837 PMC9028395

[38] Oord, A. V. D., Li, Y. & Vinyals, O. Representation learning with contrastive predictive coding. *arXiv preprint*arXiv:1807.03748 (2018).

[39] Chen, T., Kornblith, S., Swersky, K., Norouzi, M. & Hinton, G. E. Big self-supervised models are strong semi-supervised learners. *Adv. Neural. Inf. Process. Syst.* **33**, 22243–22255 (2020).

